# *Legionella* Detection in Water Networks as per ISO 11731:2017: Can Different Filter Pore Sizes and Direct Placement on Culture Media Influence Laboratory Results?

**DOI:** 10.3390/ijerph17062077

**Published:** 2020-03-20

**Authors:** Osvalda De Giglio, Giusy Diella, Paolo Trerotoli, Michela Consonni, Roberta Palermo, Marina Tesauro, Pasqualina Laganà, Gabriella Serio, Maria Teresa Montagna

**Affiliations:** 1 Regional Reference Laboratory of Clinical and Environmental Surveillance of Legionellosis, Department of Biomedical Science and Human Oncology, Section of Hygiene, University of Bari Aldo Moro, 70124 Bari, Italy; osvalda.degiglio@uniba.it (O.D.G.); giusy.diella@uniba.it (G.D.); 2 Department of Biomedical Science and Human Oncology, University of Bari Aldo Moro, 70124 Bari, Italy; paolo.trerotoli@uniba.it (P.T.); gabriella.serio@uniba.it (G.S.); 3 Department of Biomedical Surgical and Dental Sciences, University of Milan, 20133 Milan, Italy; michela.consonni@unimi.it (M.C.); marina.tesauro@unimi.it (M.T.); 4 Department of Biomedical and Dental Sciences and Morphofunctional Imaging, University of Messina, 98125 Messina, Italy; r.palermo91@gmail.com; 5 Regional Reference Laboratory of Clinical and Environmental Surveillance of Legionellosis, Department of Biomedical and Dental Sciences and Morphofunctional Imaging, University of Messina, 98125 Messina, Italy; plagana@unime.it

**Keywords:** membrane filters, *Legionella*, ISO 11731:2017, environmental surveillance

## Abstract

Determination of *Legionella* concentrations in water networks is useful for predicting legionellosis risks. The standard culture technique using concentration with membranes filters is the most commonly used method for environmental surveillance of *Legionella*. The aim of this study was to verify whether filtration with different filter pore sizes (0.2 and 0.45 µm) according to (ISO) 11731:2017, followed by directly placing them on culture media, can influence *Legionella* detection. Three laboratories participated in an experimental study that tested a known suspension of *Legionella pneumophila* (*Lpn*) serogroup 1 (ATCC 33152) (approximate final cell density of 15 CFU/mL). *E. coli* (ATCC 11775) and *Pseudomonas aeruginosa* (ATCC 25668) were included as control tests. The average (95% CI) percentage of recovery of *Lpn* was 65% using 0.45-µm filters and 15% using 0.2-µm filters (*p* < 0.0001). For control tests, the average (95% CI) percentage of recovery was higher with 0.45 vs. 0.2 µm filters: 97% vs. 64% for *Escherichia coli* (*p* < 0.00001) and 105% vs. 97% (*p* = 0.0244) for *P. aeruginosa*. Our results showed that the 0.45-µm filters provided the greatest detection of *Legionella*. Because the current national guidelines leave the choice of membrane porosity to the operator, experimental studies are important for directing operators towards a conscious choice to standardize *Legionella* environmental surveillance methods.

## 1. Introduction

Legionnaires’ disease (LD) is a severe and potentially lethal pneumonia caused by the inhalation of aerosolized water from natural and artificial water systems contaminated with *Legionella* [1,2,3,4,5,6,7,8,9]. The disease may be community-acquired or nosocomial and can occur as both sporadic and epidemic cases. LD is a growing health problem worldwide, and reported incidence rates for the disease are increasing [10,11]. In 2018, according to the National Surveillance System for LD [12], 2964 legionellosis cases were reported in Italy (incidence rate = 48.9 cases per 1 million inhabitants), which was an increase of approximately 3% compared with the previous year.

In 2015, the Italian Institute of Health updated the national guidelines for the control and prevention of legionellosis [13], highlighting that a well-designed clinical-environmental surveillance program is important for identifying sources of infection (e.g., cooling evaporative towers, water distribution systems, thermal pools, and dental units) [14,15]. Thus, laboratory investigations based on the correct enumeration of *Legionella* in water networks are necessary for validating control measures and the effectiveness of disinfection interventions [9].

The most commonly used method for the environmental surveillance of *Legionella* is the standard culture technique, which allows the estimation of the number of bacteria present in the water network. In Italy, the National Guidelines recommend the use of the International Organization for Standardization (ISO) 11731:2017 culturing method for *Legionella* enumeration for many types of water samples, including potable, industrial, waste, and natural water samples. Three different methods using selective culture media are provided and selected depending on the origin/characteristics of the water sample: i) direct plating; ii) direct placement of cellulose nitrate or mixed cellulose ester (MCE) membranes (0.2 or 0.45 µm pore size), and iii) concentration and elution with polycarbonate or polyethersulfone membrane filters (0.2 µm pore size) [16]. Direct plating of a water sample (0.1–0.5 mL) is used only when a low concentration of interfering microorganisms and a high concentration of *Legionella* species are expected (e.g., potable water). Direct placement on culture media or concentration and elution procedures with membrane filters are used when a low concentration of interfering microorganisms and a low concentration of *Legionella* species are expected.

Because the number of *Legionella* cells in any given water sample is unknown, concentration techniques are usually performed [17], and the accuracy, precision of recovery, and analytical sensitivity of various concentration techniques are well-documented in the literature [18,19]. However, the literature data regarding the direct placement of different membranes on culture media are limited and dated [20,21]. This is supported by the fact that the direct membrane placement method is relatively less sensitive than the other two methods reported in ISO 11731:2017 (direct plating of sample and concentration and elution technique) and there are issues with the recovery of certain *Legionella* species (e.g., *L. anisa*) [16].

The aim of this multicenter study was to verify whether the filtration of water with different membranes filter pore sizes and direct placement on culture media can influence the laboratory investigations for *Legionella* detection in a water network.

## 2. Materials and Methods

### 2.1. Legionella Detection

Three laboratories at different Italian Universities (C1, C2, and C3) were asked to prepare a suspension of 0.5 McFarland (approximate cell density of 1.5 × 10^8^/mL) of *Legionella pneumophila* (*Lpn*) serogroup (sg) 1 (ATCC 33152). One milliliter of 0.5 McF solution was transferred to a test tube containing 9 mL of sterile distilled water (SDW) to provide a stock suspension of 10^7^ CFU/mL. Different decimal dilutions were prepared to achieve a final cell density of approximately 15 CFU/mL on double series.

Ten milliliters of each final dilution were vortexed for 10 s and then poured into a sterile container with 90 mL of SDW (final volume = 100 mL). Each 100-mL of final dilution was filtered with a sterile MCE membrane filter, 47 mm in diameter (Millipore Corporation, Bedford, MA, USA), with a pore size of 0.2 and 0.45 µm, respectively. Each membrane filter was then transferred to a buffered charcoal yeast extract (BCYE) agar plate with cysteine (90 mm diameter), incubated at 36 ± 2 °C in a humid atmosphere (to prevent desiccation of the plates) and examined after 3, 6, and 10 days of incubation (*Legionella* grows slowly and can be masked by the growth of contaminating microorganisms). After 10 days of incubation, suspected colonies were subcultured on BCYE agar with and without cysteine (Liofilchem Srl, Teramo, Italy). Only colonies grown on BCYE agar with cysteine were subsequently identified by means of an agglutination test (Legionella latex test; Oxoid Spa, Milan, Italy). The number of colony-forming units was expressed in CFU/100 mL.

Additionally, 1 mL of the same final dilution was directly inoculated and spread on buffered charcoal yeast extract (Liofilchem Srl, Teramo, Italy) agar with cysteine as streak plate controls (140 mm diameter) to verify the concentration of the bacterial suspension to be filtered.

Control tests used standard strains of *Escherichia coli* (ATCC 11775) and *Pseudomonas aeruginosa* (ATCC 25668). The estimation of *E. coli* was performed using Chromogenic Coliform Agar, CCA (Biolife Italiana srl, Milan, Italy). Plates were incubated for 21 ± 3 h at 36 ± 2 °C, and blue colonies were counted as *E. coli* [22]. The estimation of *P. aeruginosa* was performed using Pseudomonas CN Agar plates (Microbiol & C, UTA, Cagliari, Italy). After incubation at 36 ± 2 °C for 44 ± 4 h, blue-green pyocyanin producing colonies were identified as *P. aeruginosa* [23]. The number of colony-forming units was expressed in CFU/100 mL for both microorganisms.

Each laboratory repeated the experiment of direct placement of membrane filters 15 times, for a total of 45 replicates for each microorganism, *Lpn* sg 1, *E. coli* and *P. aeruginosa*. At the same time, 15 control streak plates were generated from each laboratory for each microorganism.

### 2.2. Statistical Analysis

To evaluate the “conformity” of the number of colonies on each plate, Table B.4 Annex B ISO 8199:2018 was used as a reference [24]. If the count was within the limits suggested by the table, a Y/N flag was used for each plate, and then the total number and percentage of plates judged as “conforming” were determined for each membrane filter and laboratory. Comparisons between independent groups were performed using the chi-square test, while the concordance between membrane filters was evaluated by Cohen’s K and McNemar tests.

To compare the effects of membrane filters, a generalized linear model was used; the dependent variable was the bacterial count, and independent variables were filters, laboratories, and their interactions. The variable count was assumed to be a Poisson distribution and was summarized as a geometric mean and its 95% confidence interval. To evaluate percentage of recovery (with respect to the mean count of CFU of each microorganism grown on control streak plates generated from each laboratory), a generalized linear model was applied, which assumed the variable was normally distributed, and the assumption was verified by Shapiro–Wilk test. Factors were filters, laboratories, and their interactions. Results were shown as mean and 95% confidence interval of the mean. All post hoc comparisons were adjusted according to Tukey.

A *p*-value < 0.05 was considered statistically significant. All analyses were performed in SAS 9.4 (SAS Institute Inc., Cary, NC, USA) for personal computers.

## 3. Results

The final solution streaked on the control plate was found to contain a mean count of 15 CFU/mL (11–19 CFU/mL) of *Lpn* sg 1. Conformity was achieved in 86.7% (39/45) of the plates from 0.45-µm filters and in 0% (0/45) of the plates from 0.2-µm filters (*p* < 0.0001). The analysis of concordance by the McNemar test between the filters showed a lack of concordance (*p* < 0.0001) and a coefficient K = 0 suggesting non-concordance between the membrane filters.

The mean count (95% CI) for *Lpn* sg 1 was 9.68 CFU/100 mL (8.82–10.65 CFU/100 mL) for 0.45-µm filters and 2.22 CFU/100 mL (1.83–2.71 CFU/100 mL) for 0.2-µm filters. The model showed a statistically significant difference between the two filters (*p* < 0.0001), but there was no statistically significant difference among laboratory centers (*p* = 0.1004). In addition, the interaction was not statistically significant (*p* = 0.257).

Table 1 shows all count comparisons (95% CI) and recovery percentages (%) between laboratories and filters with adjusted *p*-values. Except for the mean count of C1 and C3 with 0.45-µm membrane filters, there were no other statistically significant comparisons. The average (95% CI) percentage of recovery was 65% (62% to 68%) using 0.45-µm membrane filters and 15% (12% to 18%) using 0.2-µm membrane filters (*p* < 0.0001). The average percent recoveries were statistically significant different between laboratories (*p* < 0.0001); in addition, interactions between laboratories and membranes were statistically significant (*p* < 0.0001). In fact, in each laboratory, there was a statistically significant difference between 0.45- and 0.2-µm membrane filters (Table 1).

In the control tests, the final solution streaked on the control plate was found to contain a mean count of 6 CFU/mL (4–9 CFU/mL) of *E. coli* and 13 CFU/mL (10–19 CFU/mL) of *P. aeruginosa*. The mean counts of *E. coli* and *P. aeruginosa* were all within the conformity limits at all laboratories and with both types of membrane filters (0.2- and 0.45-µm). For *E. coli*, the mean count (95% CI) was 5.78 CFU/100 mL (5.11–6.53 CFU/100 mL) with the 0.45-µm pore size filters and 3.6 CFU/100 mL (3.07–4.23 CFU/100 mL) with the 0.2-µm pore size filters (*p* < 0.0001).

Table 2 shows the results of the count comparisons (95% CI) and recovery percentages (%) of *E. coli* by laboratories and membranes. For mean counts, there was a statistically significant difference among membrane filters only in C3 (*p* < 0.0001) and among laboratories only for 0.2-µm filters (*p* < 0.0001). The average (95% CI) percent of recovery for *E. coli* was 64% (59%–68%) with 0.2-µm filters and 97% (93%–100%) with 0.45-µm filters; this difference was statistically significant (*p* < 0.00001). The model showed a statistically significant effect of laboratories between membrane filters (*p* < 0.0001) and for interactions (*p* < 0.0001), as shown in Table 2.

The count averages and percentages of recovery for *P. aeruginosa* are shown in Table 3. The model for counts did not show statistically significant differences between filters or among laboratories. However, the analysis of percentage (%) of recovery showed that the average (95% CI) was 97% (92–101%) with 0.2-µm filters, while it was 105% (101%–109%) with 0.45-µm filters; this difference was statistically significant (*p* = 0.0244). There was a significant effect of membrane filters for only C2 (*p* = 0.024) and of laboratories (*p* = 0.0026) on the recovery averages.

## 4. Discussion

To date, there is very little and dated literature comparing the effects of different membrane pore sizes on the recovery of microorganisms after filtration and direct placement on the culture media. These studies have focused on the *Pseudomonas* and coliform genera [20,21].

To the best of our knowledge, our multicenter study is the first carried out with *Legionella* aimed at comparing MCE membranes with different pore sizes (0.2- and 0.45-µm) by the direct placement method on culture media. Some previous researchers compared the recovery of membranes of different materials and pore sizes for *Legionella* spp. with the concentration and elution method, detecting the highest percentage of recovery with 0.2-µm vs. 0.45-µm pore-size filters [19,25].

Although the ideal characteristics of a membrane filter for the quantitative analysis of bacteria were assumed to be pores small enough to retain bacteria, our findings showed that all three laboratories in the study detected the non-compliant mean count of *Lpn* using 0.2-µm filters and greater recovery using 0.45-µm filters. Several hypotheses can explain our data. Previous research stated that 0.45-µm filters would allow a greater diffusive transfer of nutrients and maintain the cells in a more hydrated environment, leading to a higher recovery efficiency [21]. In addition, it has been shown that at least some species of bacteria, when physically damaged or metabolically stressed, are more efficiently detected using a membrane with a relatively open pore structure [20,21].

Other researchers [26,27,28,29] observed that during filtration, some microorganisms (e.g., bacteria, yeasts, and viruses) that were smaller than the membrane pores were retained in considerable quantities by adhering to the membrane texture. Thus, microorganisms smaller than the filter pores, which one would expect to pass through the pores, have most likely remained trapped within the filter due to electrostatic forces rather than mechanical phenomena. In addition, to analyze samples that are difficult to filter for the turbidity, some authors and guidelines have recommended using membranes with larger pores to prevent clogging or reduced flow [16,25,30] that would reduce the microorganism recovery by the filter.

These hypotheses could also be applied to *Legionella*, a microorganism of 0.3–0.9 µm in width that is retained by 0.2-µm filters by mechanical action but adheres and is trapped in the meshes of 0.45-µm filters by electrostatic forces. The 0.45-µm membrane pore size allows *Legionella* to have a greater contact with nutrients present in the culture medium, allowing for maximum recovery. Moreover, with 0.2-µm pore-size membranes, the occlusion of the pores by *Legionella* could occur more easily and in such a way as to prevent the development of colonies on the culture medium.

During the control tests, *E. coli* (1 µm in width) and *P. aeruginosa* (0.5–1 µm in width) showed conformity for the mean count for both 0.2- and 0.45-µm filters. Similar to *Legionella*, *E. coli* and *P. aeruginosa* showed a higher average percentage of recovery with 0.45- vs. 0.2-µm filters in other studies that focused on fecal coliforms [21]. A limitation of our study is that the percentage of recovery for *E. coli* was affected by the very low count on the control plate (6 CFU/mL). Therefore, a minimum difference of CFU between membranes with different pore sizes or among laboratories turns into a very large margin that could bias the interpretation of results.

Overall, the results of the control tests showed that the behavior of *Legionella* did not seem related to the cell size of the microorganism but to its interaction with the membrane. Therefore, future studies using scanning electron photomicrographs to visualize the way in which bacteria penetrate into the different membrane pore sizes and remain attached to them should be conducted.

## 5. Conclusions

The ideal characteristic of a membrane filter for the quantitative analysis of bacteria appears to be pores small enough to retain bacteria. Given our results showing greater recovery with the membranes of 0.45-µm pore sizes compared to 0.2-µm pore sizes, it is reasonable to conclude that 0.45-µm filters provide the greatest assurance of detecting *Legionella*.

Considering that the current national guidelines leave the choice of membrane porosity (0.2 or 0.45 µm) to the operator, increasing the number of experimental studies on the direct placement of the membrane on culture media is important. These experiments will help direct operators towards a conscious choice of membrane porosity and improve the standardization of the *Legionella* environmental surveillance methods in the laboratory and lead to improved comparison of the results.

## Figures and Tables

**Table 1:** *Legionella pneumophila* sg 1 experiment: averages and 95% confidence intervals for each laboratory and type of membrane filter. *p*-values from the generalized model were adjusted according to Tukey.

Pore Size Filter	0.45 µm	0.2 µm	0.45 µm vs. 0.2 µm
Laboratories	*L. pneumophila* sg 1 counts (CFU/100 mL)		*p*-Value
C1	12.13 (10.49 to 14.03)^1^	2.27 (1.62 to 3.17)	<0.0001
C2	9.07 (7.66 to 10.73)	2.6 (1.89 to 3.56)	<0.0001
C3	8.27 (6.93 to 9.86)^1^	1.87 (1.29 to 2.7)	<0.0001
Percentage (%) of recovery			
C1	81 (76 to 86)^2,3^	15 (10 to 20)	<0.0001
C2	60 (55 to 65)^2^	17 (12 to 20)	<0.0001
C3	55 (50 to 60)^3^	12 (7 to 17)	<0.0001

In 0.45 µm: ^1^ C1 vs. C3 *p* = 0.0126, ^2^ C1 vs. C2 *p* < 0.0001, ^3^ C1 vs. C3 *p* < 0.0001.

**Table 2:** *Escherichia coli* experiment: averages and 95% confidence intervals for each laboratory and type of membrane filter. *p*-values from the generalized model were adjusted according to Tukey.

Pore Size Filter	0.45 µm	0.2 µm	0.45 µm vs. 0.2 µm
Laboratories	*E. coli* counts (CFU/100 mL)		*p*-Value
C1	5.47 (4.04 to 6.79)	4.23 (3.34 to 5.45)^1^	0.6734
C2	6.53 (5.36 to 7.96)	5.13 (4.11 to 6.42)^2^	0.6094
C3	5.4 (4.34 to 6.71)	2.13 (1.51 to 3.02)^1,2^	0.0001
Percentage (%) of recovery			
C1	91 (84 to 98)^3^	71 (64 to 78)^5^	0.0016
C2	109 (102 to 116)^3,4^	86 (78 to 93)^6^	0.0001
C3	90 (83 to 97)^4^	36 (28 to 43)^5,6^	<0.0001

In 0.2 µm: ^1^ C1 vs. C3 *p* = 0.0172, ^2^ C2 vs. C3 *p* = 0.0004; in 0.45 µm: ^3^ C1 vs. C2 *p* = 0.0081, ^4^ C2 vs. C3 *p* = 0.0037; in 0.2 µm: ^5^ C1 vs. C3 *p* < 0.0001, ^6^ C2 vs. C3 *p* = 0.0081.

**Table 3:** *Pseudomonas aeruginosa* experiment: averages and 95% confidence interval for each laboratory and type of membrane filter. *p*-values from the generalized model are adjusted according Tukey.

Pore Size Filter	0.45 µm	0.2 µm	0.45 µm vs. 0.2 µm
Laboratories	*P. aeruginosa* counts (CFU/100 mL)		*p*-value
C1	12.4 (10.74 to 14.32)	11.8 (11.18 to 13.67)	0.9971
C2	14.67 (12.85 to 16.74)	11.93 (10.31 to 13.82)	0.3146
C3	14 (12.23 to 16.03)	14.07 (12.29 to 16.09)	1
Percentage (%) of recovery			
C1	95 (87 to 103)^1^	91 (83 to 99)^2^	0.966
C2	113 (105 to 121)^1^	92 (84 to 99)^3^	0.0031
C3	108 (99 to 115)	108 (100 to 103)^2,3^	1

In 0.45 µm: ^1^ C1 vs. C2 *p* = 0.0272; in 0.2 µm: ^2^ C1 vs. C3 *p* = 0.0272; ^3^ C2 vs. C3 *p* = 0.0463.
